# Annealing induced a well-ordered single crystal δ-MnO_2_ and its electrochemical performance in zinc-ion battery

**DOI:** 10.1038/s41598-019-51692-x

**Published:** 2019-10-22

**Authors:** Ryan Dula Corpuz, Lyn Marie Z. De Juan, Supareak Praserthdam, Rojana Pornprasertsuk, Tetsu Yonezawa, Mai Thanh Nguyen, Soorathep Kheawhom

**Affiliations:** 1 Department of Chemical Engineering, Faculty of Engineering, Chulalongkorn University, Bangkok, 10330 Thailand; 2 Department of Physics, School of Science and Engineering, Ateneo de Manila University, Quezon City, 1108 Philippines; 3 Department of Chemical Engineering, Faculty of Engineering, University of Santo Tomas, Manila, 1015 Philippines; 4 High-performance computing unit (CECC-HCU), Center of Excellence on Catalysis and Catalytic Reaction Engineering (CECC), Chulalongkorn University, Bangkok, 10333 Thailand; 5 Department of Materials Science, Faculty of Science, Chulalongkorn University, Bangkok, 10330 Thailand; 6 Research Unit of Advanced Materials for Energy Storage, Chulalongkorn University, Bangkok, 10330 Thailand; 7 Center of Excellence in Petrochemical and Materials Technology, Chulalongkorn University, Bangkok, 10330 Thailand; 8 Division of Materials Science and Engineering, Faculty of Engineering, Hokkaido University, Kita 13 Nishi 8, Sapporo, Hokkaido, 060-8628 Japan

**Keywords:** Batteries, Chemical engineering, Batteries

## Abstract

Herein, the formation and electrochemical performance of a novel binder-free turbostratic stacked/ well-ordered stacked δ-MnO_2_-carbon fiber composite cathodes in deep eutectic solvent (DES) based zinc-ion battery (ZIB) is reported. Results of morphological, elemental, and structural analyses revealed directly grown and interconnected *δ*-MnO_2_ crumpled nanosheets on a carbon fiber substrate. Moreover, an improvement via a simple annealing strategy in the stacking, surface area and conductivity of the *δ*-MnO_2_ sheets was observed. Annealing induces the rearrangement of *δ*-MnO_2_ sheets resulting in the transformation from turbostratic stacking to a well-ordered stacking of ${\delta }$-MnO_2_ sheets, as indicated by the selected area electron diffraction (SAED) hexagonal single crystal pattern. Besides, the formation of the well-ordered stacking of ${\delta }$-MnO_2_ sheets exhibited improved electrochemical performance and cyclability, as cathode material for ZIB. The novel strategy described in this study is an essential step for the development of binder-free δ-MnO_2_-C fiber composite with a well-ordered stacking of *δ*-MnO_2_ sheets. This study also demonstrated comparable electrochemical performance between the turbostratic ${\delta }$-MnO_2_ sheets and the well-ordered stacked *δ*-MnO_2_ sheets.

## Introduction

Nowadays, a zinc-ion battery (ZIB) is hotly pursued by researchers. This is due to its interesting properties as energy storage material with potential applications in rechargeable batteries as used in laptops, cellphones, wireless electronic gadgets, implantable medical devices etc^1–7^. This renewed interest in ZIB has been witnessed during the past decade, especially with the advent of nanoscience and technology. Predominantly, novel *ex-situ* and *in situ* spectroscopic techniques, advanced, and sophisticated tools, such as high-resolution transmission electron microscopy (HR-TEM), scanning electron microscopy (SEM), X-ray diffraction (XRD), X-ray photoelectron spectroscopy (XPS) etc. for characterization of nano-dimensions, were developed. This was done in order to understand better its structure-property relationships and the need to produce novel materials that could address the demand for economical, efficient, environment-friendly, and safe energy storage materials^8,9^.

A ZIB typically consists of zinc (Zn) anode which exhibits a theoretical capacity of 820 mAhg^−1^. Zn is relatively abundant, environmental friendly, economical, and safe compared to lithium (Li)^10–13^. Recently, among the cathode materials paired with Zn anode, growing interest in MnO_2_ cathode has been reported. Possibly, this is because MnO_2_, like Zn, is likewise plentiful. It is evident that MnO_2_ has a high theoretical capacity of about 308 mAhg^−1^. In addition, it has well-studied phases: $\alpha ,\beta ,\delta ,\text{and}\,\gamma$-MnO_2_, well-defined morphologies: nanourchin, nanorods, nanosphere, nanosheets, nanoflakes, nanocorals, nanoflowers, and nanowires, interesting nanostructures: 1D, 2D, and 3D self-assembled into varieties of forms such as tunnels, sheets etc. which could serve as intercalation type electrode for ZIBs. Further, MnO_2_ has ease of synthesis via common scalable routes: electrodeposition, chemical reduction, thermal decomposition, and hydrothermal methods^14,15^.

Although Zn-MnO_2_ based batteries are well-documented, most of the literature published in this field has been typically reported under aqueous^16–19^ and non-aqueous^20,21^ electrolyte systems. Accordingly, a previous paper had investigated the deep eutectic solvent (DES) based electrolyte system and applied it to Zn-MnO_2_ secondary battery^22^. DES^23,24^, which is typically formed from the eutectic mixture of Lewis or Bronsted acids and bases, is interesting for the fact that H^+^ ion intercalation and electrolysis of water (hydrogen evolution) can be avoided. Besides, anode passivation and formation of ZnO and dendrites commonly observed in aqueous systems could be prevented. This development will eventually make Zn^2+^ intercalation as the governing route to understand the complex interaction mechanism of divalent cations with MnO_2_ cathode. An understanding of the said mechanism is not only important experimentally but also important for simulation and theoretically researches whereby improvement can be undertaken to boost the electrochemical performance of ZIBs.

At present, a common obstacle encountered in using MnO_2_ cathode is its poor conductivity^25^ which could increase the internal resistance of the ZIBs resulting in poor electrochemical performance. A remedy frequently used by researchers is the addition of conductive materials such as carbon black^26^, reduced graphene oxide (RGO)^27^, conducting polymer^28^ and carbon nanotubes (CNTs)^29^ usually with the aid of polymeric binder to enhance the adhesion of the additives and active material on the current collector. However, this not only complicates the system but also makes the cost of producing the ZIBs expensive. In this study, a novel strategy was developed to address the poor conductivity of MnO_2_ as well as the undesirable corrosion problem which is widely encountered in aqueous based ZIBs. A first approach, based on direct deposition and growth of active material MnO_2_ on a conductive carbon fiber, was to synthesize a binder-free MnO_2_-C composite cathode via an economical hydrothermal process. This method not only eliminated the need for expensive polymer binders, but most importantly improved the adhesion and connectivity of the active materials to the substrate which is necessary for efficient charge transfer. Secondly, the introduction of an annealing technique was undertaken to enhance δ-MnO_2_ sheets stacking order, surface area, and conductivity of the active materials. This was done to generate more redox and intercalation sites for charge storage. Further, it was found that annealing at 300 °C for 1 h can induce the transformation from turbostratic stacking to a well-ordered stacking of δ-MnO_2_ sheets. Hence, in a review of the literature, it would appear that this study is the first to analyze the transformation from turbostratic stacking to a well-ordered stacking of δ-MnO_2_ sheets via annealing, and its effect on the electrochemical performance of δ-MnO_2_, as cathode material for ZIB utilizing DES as electrolyte.

## Experimental

### Materials

All chemicals were used as received without further purification: potassium permanganate (KMnO_4_, QReC), ammonium sulfate ((NH_4_)_2_SO_4_, Sigma-Aldrich), urea (Ajax Finechem), choline chloride (Sigma-Aldrich), zinc chloride (ZnCl_2_, Ajax Finechem), zinc sulfate (ZnSO_4_, Ajax Finechem), carbon cloth (AvCarb 1071 HCB, AvCarb Material Solutions), deionized water, isopropyl alcohol (IPA, Ajax Finechem) and nickel foam (99.97%, 100 pores per inch (PPI) and 1 mm thick, Qijing Trading Co., Ltd.), sulfuric acid (H_2_SO_4_, Ajax Finechem).

### Substrate preparation

The carbon cloth was surface treated with 1.0 M sulfuric acid for 1 h, washed with DI water several times, and vacuum dried at 60 °C for 2 h before usage.

### Cathode synthesis and fabrication

In a typical experiment, 0.1264 g KMnO_4_ and 0.0428 g (NH_4_)_2_SO_4_ were dissolved and mixed in 40 mL deionized water. The resulting solution was then sonicated for 1 h and hydrothermally synthesized at 110 °C for 18 h using Teflon lined autoclave decorated with carbon cloth on its inner wall. Next, the carbon cloth, deposited with MnO_2_ particles, was washed with deionized water several times and rinsed with IPA. Finally, the fabricated cathode was vacuum dried for 2 h and annealed for 1 h at 300 °C with a heating rate of 10 °C/min.

### Anode synthesis and fabrication

In a typical experiment, Nickel foam was used as a substrate immersed in 1.0 M ZnSO_4_ solution to deposit Zn via electrodeposition. The applied voltage was set at 1.4 V for 1.5 h.

### DES synthesis and electrolyte preparation

In a typical experiment, 50 g choline chloride and 43 g urea were mixed together inside a glove box until a homogenous solution was achieved. Then, 4.1 g ZnCl_2_ was added to the mixture and thoroughly mixed overnight.

### ZIB assembly

CR2032 cell was used to assemble the ZIB. The assembled battery typically consisted of an anode, cathode, and electrolyte made up of Zn electrodeposited on Ni foam. Further, MnO_2_ hydrothermally was grown on carbon cloth, and ZnCl_2_ electrolyte was dissolved in DES, respectively. The electrodes were separated with microfiber glass and were enclosed within circular metal cases. To prevent the electrode from moving around, a metal spacer and spring were placed adjacent to the anode and the negative case, respectively, as shown in Fig. S1.

### Material characterization

Both elemental analysis and morphology were investigated using scanning electron microscope (SEM, JEOL JSM-6480LV, 15 kV) and transmission electron microscope (TEM, JEOL JEM-1400, 100 kV). The crystalline and phase structure was determined using X-ray diffraction (XRD, Bruker D8-Advance, Cu Kα radiation, λ = 1.5418 Å) operating at 40 kV and 40 mA with 2θ range of (5 to 90) degrees. The surface area was determined using BET (Microtrac, BELSORP mini II).

### Electrochemical characterization

To evaluate the electrochemical performance of the fabricated Zn-MnO_2_ battery, the following electrochemical tests were conducted: cyclic voltammetry (CV), electrochemical impedance spectroscopy (EIS) and galvanostatic charge-discharge test.

1. The galvanostatic charge-discharge test was conducted using battery testing system (NEWARE) within the voltage range: (0.4–1.9) V for 10 cycles per current density (50, 100, 150, and 200) mA g^−1^.
2. The electrochemical impedance spectroscopy was conducted using VersaSTAT 3F (AMETEK) within the frequency range: (0.01–100,000) Hz.
3. The cyclic voltammetry test was conducted using VersaSTAT 3F (AMETEK) within the voltage window of (0.4–1.9) V at a scan rate of 0.005 V/s.

## Results and Discussion

The aim of this study is to synthesize a binder free MnO_2_-C fiber composite with improved conductivity without the addition of a conductive agent e.g. conductive carbon, graphene or carbon nanotube (CNT). A further objective is to investigate its electrochemical performance as a cathode material for ZIBs. To realize this, it is imperative to produce particles with uniform morphology i.e. size, shape, and structure. In so doing, a relationship among morphology, structure, and property can be established. This is important not only for basic or fundamental science but especially important for enhancing the performance of ZIBs. Hence, this study focuses on thorough optimization of the synthesis parameters viz. time and temperature which can significantly affect the resulting morphology, structure, and the performance of the battery^30–33^.

**Figure 1:**
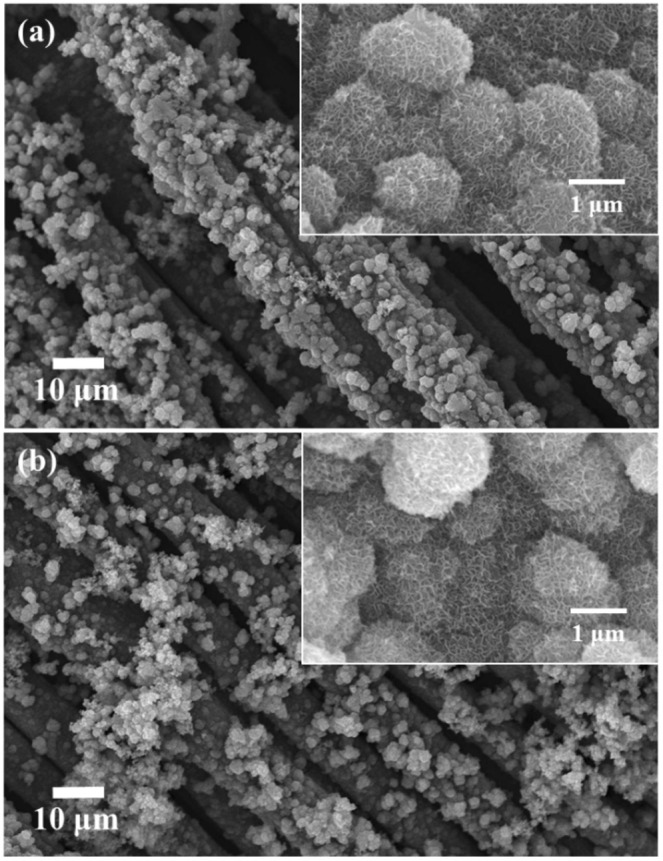
SEM micrograph of hydrothermally synthesized MnO_2_ in (**a**) un-annealed and (**b**) annealed conditions.

Figure 1 shows the morphology of samples synthesized at 110 °C for 13 h in un-annealed and annealed conditions. It can be observed that the particles hydrothermally synthesized have a flower-like morphology (Fig. 1a) which was retained even after annealing at 300 °C (Fig. 1b). EDS analysis of the samples, as shown in Fig. S2a,b, revealed the presence of elements Mn, O and K in the produced microsphere, for both un-annealed and annealed conditions which strengthen the likelihood of the existence of MnO_2_. To investigate the phase and structure of the synthesized powder, XRD analysis was done for the as-synthesized powder in its un-annealed and annealed conditions, as shown in Fig. Sα. XRD analyses revealed that the as-synthesized powder (un-annealed) had a $\delta$ phase that can be judged from the broad prominent peaks around 2*θ* = 12.636°, 25.43°, 36.551°, 54.031° and 65.432° corresponding to (200), (400), (020), (613), and (033) planes respectively of the orthorhombic $\delta$-MnO_2_ polymorph^34–40^. On the other hand, the annealed powder showed similar peaks with that of the un-annealed sample with more intense and sharper peaks at 2*θ* of ~36° and ~65°. These peaks can be attributed to the change in the stacking of δ-MnO_2_ and not to the formation of another compound since the annealing temperature employed is not enough to cause phase or structural transformation^41,42^. This phenomenon will be discussed in the following paragraph.

*δ*-MnO_2_ has a 2-D layered structure (Fig. S3b) wherein the layers typically consist of edge-sharing MnO_6_ octahedra with space in between layers which can be occupied by cations such as K^+^, Li^+^, Na^+^, other alkaline metals ions, and water molecules to stabilize the randomly distributed deficiency of charges in the structure^36^. The orthorhombic *δ*-MnO_2_ (K_0.33_MnO_2_·0.66H_2_O), was found to be in good agreement with that of the EDS elemental analysis (Fig. S2c), wherein the amount of K^+^ per Mn atom in both un-annealed and annealed samples are ~14–20% which is one of the polymorph of K_0.33_MnO_2_·0.66H_2_O^34^. The broadening of peaks that can be seen are usually attributed to small nanoparticles, amorphous, and disordered structures. These are commonly observed phenomenon in hydrothermally synthesized *δ*-MnO_2_. However, in some instances, this occurrence can also be associated with the variation in the interlayer spacing or interlayer order^34,43^.

**Figure 2:**
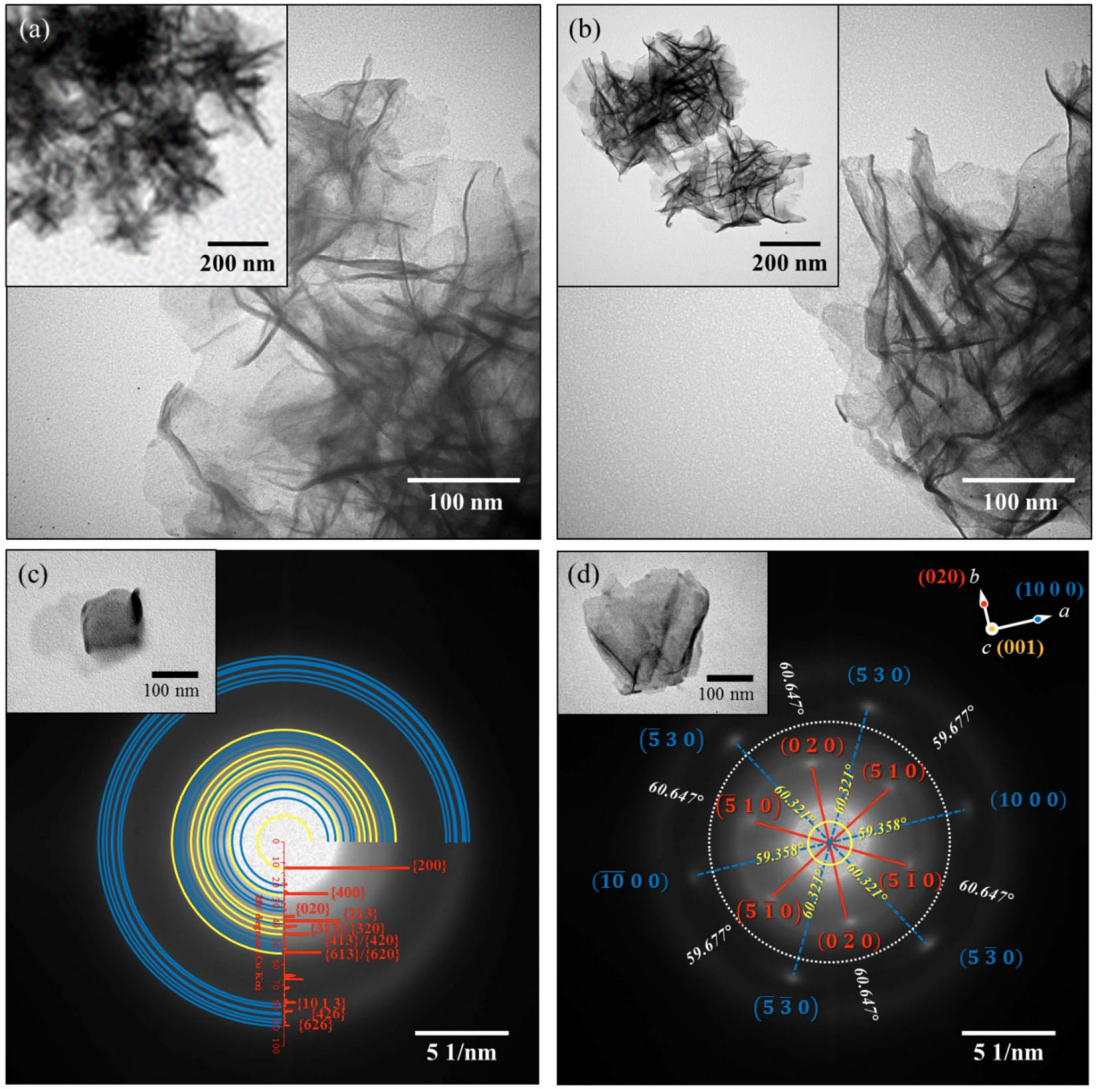
(**a**,**b**) TEM images and (**c**,**d**) SAED pattern of (**a**,**c**) un-annealed and (**b**,**d**) annealed samples. In (**a**,**b**) the inset images correspond to the low magnification images. In (**c**,**d**) the inset images indicate the TEM images of the analyzed area. In (**c**) the inset graph is the calculated XRD spectrum of orthorhombic *δ*-MnO_2_ (K_0.33_MnO_2_·0.66H_2_O)^34^.

Figure 2a,b show the TEM images of the particles formed for both un-annealed and annealed samples. It was observed at micro-level that the flower-like morphology under SEM were actually crumpled nanosheets that have a flower-like morphology. The formation of sheet-like morphology was found to be in good agreement with the Wulff-constructed equilibrium shape of *δ*-MnO_2_ (Fig. S4) wherein the surface of the nanosheets contains the low surface energy plane {001}. According to Chen *et al*.^34^, this structure had an average surface energy of 0.11 Jm^−2^ and an atomic volume of 32.9 Å^3^/Mn atom. The sheet-like morphology was examined under SAED, as shown in Fig. 2c,d. It was observed that the un-annealed sample had a polycrystalline structure which is indexed to the planes of the orthorhombic *δ*-MnO_2_. The annealed sample exhibited single crystal diffraction which is indexed to {020} and {510} for the inner hexagonal pattern and {530} and {10 0 0} for the outer hexagonal pattern. These were in good agreement with the XRD peaks, wherein the annealed sample exhibited sharp and intense peaks at 2θ = 36.551°, 36.923°, 66.041°, and 66.769° which correspond to {020}, {510}, {530} and {10 0 0}, respectively. The absence of the hexagonal single crystal pattern for the un-annealed sample can be attributed to the large degree of disorder, i.e. turbostratic stacking of MnO_2_ sheets. This observation well agreed with the obtained broadening of XRD peaks for the un-annealed sample along with the results obtained by other researchers^34,43^. Hence, after annealing, the hexagonal single crystal pattern SAED profile indicated that the well-ordered MnO_2_ structure was obtained.

**Figure 3:**
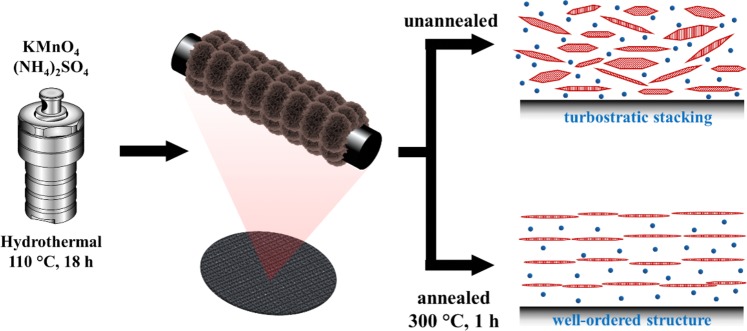
Formation of orthorhombic *δ*-MnO_2_ via hydrothermal method and the transition from turbostratic stacking to well-ordered structure of orthorhombic $\delta$-MnO_2_ via annealing. The red hexagonal disk and blue circles correspond to the sheet-like MnO_2_ and surface water, respectively.

Figure 3 illustrates the formed particles before and after annealing. Hydrothermal synthesis at 110 °C for 13 h utilizing KMnO_4_ and (NH_4_)_2_SO_4_, produced crumpled sheet-like morphology of orthorhombic δ-MnO_2_, which were directly grown on the carbon current collector in the absence of any polymeric binder. These particles have a high degree of disordered stacking i.e. turbostratic stacking, as indicated by the circular rings (polycrystalline) in the SAED analysis and the broad XRD peaks. However, these disordered structures realigned to form a well-ordered structure after annealing at 300 °C for 1 h. This phenomenon was clearly observed through the transition from the polycrystalline SAED pattern of un-annealed to the hexagonal single-crystal SAED pattern of the annealed sample.

**Figure 4:**
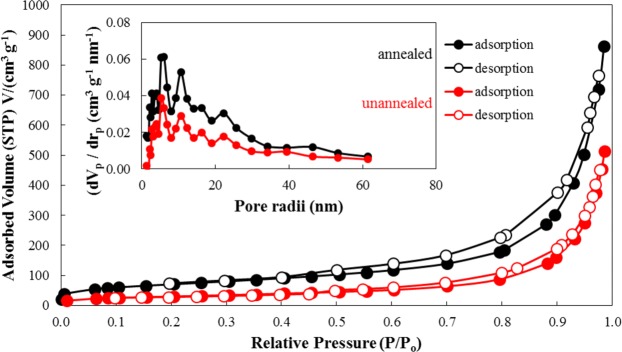
BET N_2_ adsorption-desorption isotherm, with inset of BJH pore size distribution.

**Figure 5:**
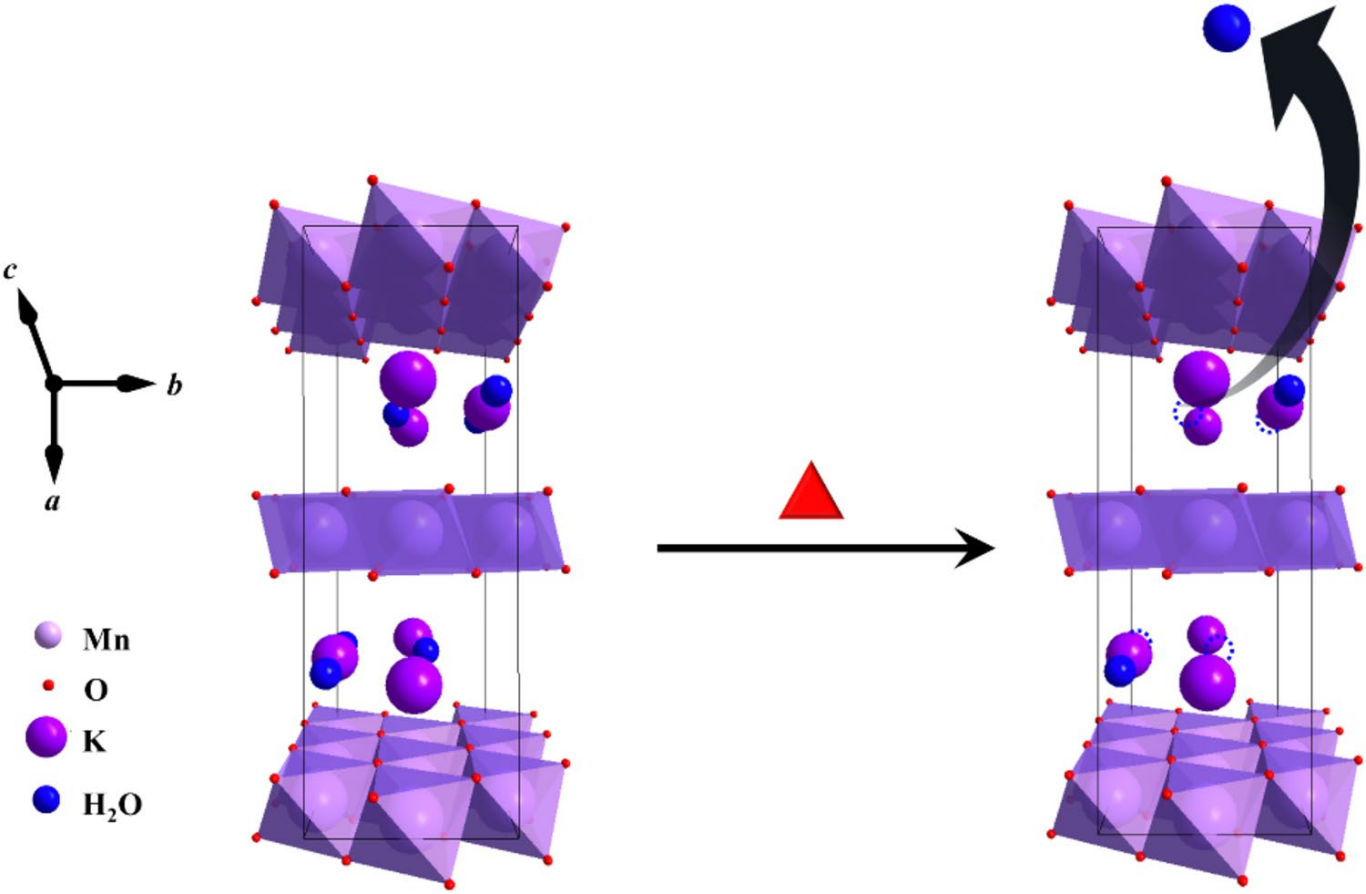
Schematic illustration of the proposed mechanism for the increase in the BET surface area of *δ*-MnO_2_ after annealing. The structure corresponds to a single unit cell of orthorhombic *δ*-MnO_2_.

As shown in Fig. 4 and Table S1, BET analysis and pore size distribution of the un-annealed and annealed MnO_2_ were measured. This was carried out to determine the change in the surface area upon the transition from turbostratic stacking to well-organized stacking. The adsorption-desorption of both the un-annealed (turbostratic stacking) and annealed (well-ordered stacking) sample exhibits Type II isotherm, as shown by the large measured C value (BET) in Table S1. Both samples also follow an H_3_ hysteresis loop in the range (0.5 to 1.0) *P/P*_*o*_ and (0.4 to 1.0) *P/P*_*o*_ for un-annealed (turbostratic stacking) and annealed (well-ordered stacking) samples, respectively. According to IUPAC classification, this indicates the presence of sheet-like particles forming slit-shaped pores^44^. Computation of its corresponding BET surface area revealed that the annealed δ-MnO_2_ (well-ordered structure) had a surface area equal to 246.18 m^2^ g^−1^ greater than the surface area of the un-annealed δ-MnO_2_ (turbostratic stacking) which was around 106.05 m^2^ g^−1^. Moreover, the pore volume (1.3188 cm^3^ g^−1^) of the annealed sample increased in comparison to the pore volume of the un-annealed sample (0.7776 cm^3^ g^−1^). The increase in the BET surface area and BJH pore volume after annealing can be attributed to the removal of adsorbed and interlayer water molecules, as illustrated in Fig. 5. Hereafter, the term annealed and un-annealed is used to represent well-ordered stacking and turbostratic stacking, respectively, for simplicity.

**Figure 6:**
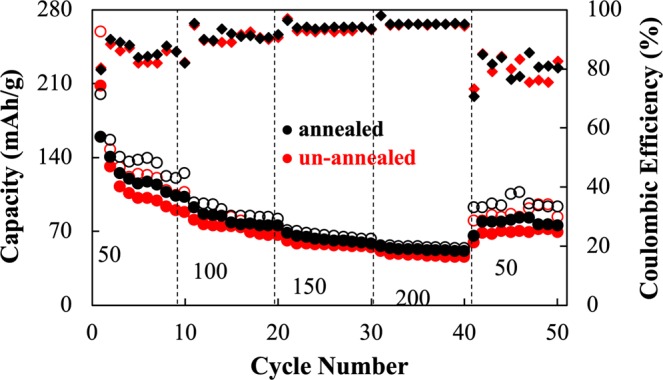
Galvanostatic charge-discharge capacity of the un-annealed (turbostratic stacking: red) and annealed (well-ordered stacking: black) samples at different current densities. The hollow and filled circles correspond to charge and discharge values respectively, while diamond corresponds to the coulombic efficiency data.

Figures 6 and S5, representing the galvanostatic charge-discharge capacity profile, show the galvanostatic charge-discharge capacity at different current densities. It was observed that the un-annealed sample exhibited a higher initial discharge capacity of about 208 mAhg^−1^ in comparison to the annealed sample having an initial discharge capacity of 158 mAhg^−1^. However, during cycling, in the 2^nd^ and 10^th^ cycle, the discharge capacity of the un-annealed sample drastically dropped to 130 mAhg^−1^ and then dropped down to 88 mAhg^−1^, respectively. As for the annealed sample, the discharge capacity did not drastically change. However, in the 2^nd^ and 10^th^ cycle, the capacity slowly dropped to 140 and 105 mAhg^−1^, respectively.

Subsequently, when the current density increased to 100 mAg^−1^, it was found that the un-annealed sample reached a plateau of around 75 and 66 mAhg^−1^. Nevertheless, the annealed sample attained a plateau at 84 and 74 mAhg^−1^. At 150 mAg^−1^, the un-annealed sample approached a plateau at around 56 mAhg^−1^ and the annealed sample at 60 mAhg^−1^. Furthermore, at 200 mAg^−1^, the un-annealed sample displayed a plateau around 45 mAhg^−1^ and the annealed one displayed a plateau around 50 mAhg^−1^. Finally, when the applied current density was returned to 50 mAhg^−1^, the un-annealed sample showed a plateau around 70 mAhg^−1^ and the annealed sample showed a higher discharge capacity plateau of 80 mAhg^−1^.

After the 50^th^ cycle (Fig. S6), both un-annealed and annealed samples showed a stable discharge capacity of around 70 mAhg^−1^ at an applied current density of 100 mAg^−1^. During the 1^st^ and 2^nd^ cycle, especially for the un-annealed sample, discharge capacity abruptly faded. This most probably happened due to irreversible structural/volumetric change^45^ during the insertion of Zn^2+^and the dissolution of the active material MnO_2_. This is a common phenomenon since MnO_2_ is somewhat soluble in DES^46^. Besides, CV information after 50^th^ cycle is shown in Fig. S9. The CV result revealed that the structure did not significantly change due to insignificant change in position of the redox peaks.

**Figure 7:**
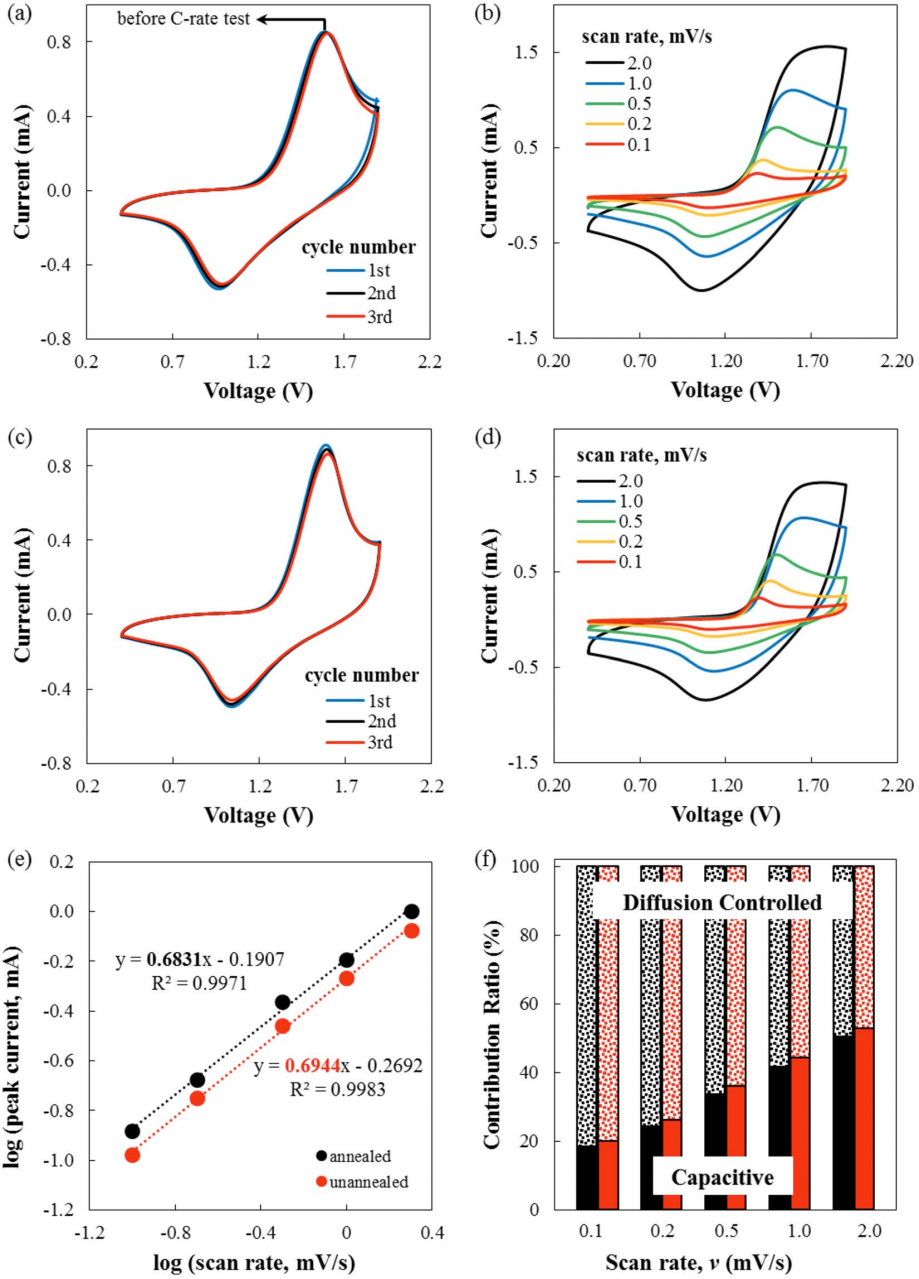
(**a**–**d**) Cyclic voltammogram of the (**a**,**b**) annealed and (**c**,**d**) un-annealed samples within the voltage window: (0.4–1.9) V vs. Zn/Zn^2+^ using (a,c) 0.5 mV/s scan rate and (**b**,**d**) multiple scan rates. (**e**) log (*i*) vs. log (*v*) of the cathodic peaks of both annealed (red) and un-annealed samples (black). (**f**) Contribution ratio of capacitive and diffusion-controlled capacity at different scan rates for both annealed (black) and un-annealed (red) samples.

Figure 7 shows the cyclic voltammogram of un-annealed and annealed samples within the voltage window: (0.4–1.9) V measured at a scan rate of 0.5 mV/s (a, c). Prominent peaks around 1.05 V and around 1.5 V were observed which proved to be in good agreement with the results obtained by other researchers^22^. These peaks are attributed to the insertion (discharge)/extraction of Zn^2+^ (charge) on the MnO_2_ cathode during the discharging and charging of the ZIB. It can also be inferred that overlapping curves of the first 3 cycles in both un-annealed and annealed conditions (a, c) imply good cycling stability of both samples. At low scan rate (b, d), obvious peaks were observed implying redox reaction on the electrodes. However, at high scan rate, the redox peaks are no longer visible, and both samples were showing surface capacitive-like behavior.

To understand the overall governing charge storage mechanism, Eq. (1) was employed:

1 $$i(V)={k}_{1}v+{k}_{2}{v}^{1/2}$$

where *k*_1_*v* corresponds to the surface capacitive effects attributed to pseudocapacitive behavior and double layer charge storage, and ${k}_{2}{v}^{1/2}$ corresponds to the diffusion part^4,18,47–50^. Based on the histogram shown in Fig. 7f and the tabulated values of percent contribution (Table S2) obtained from the relationship given in Eq. (1), it can be assumed that the governing charge storage mechanism, for both un-annealed and annealed samples in the considered scan rates, is dominated by diffusion. This result is consistent with the *b* values obtained which are around 0.6 (Fig. 7e and Table S3) for both conditions and is therefore predominantly diffusion controlled, as in Eq. (2):

2 $$i=a{v}^{b}$$

where *a* and *b* are adjustable parameters. If *b* is approximately equal to 1, the charge storage mechanism is predominantly surface capacitive in nature, whereas if *b* is approximately equal to 0.5, then it is diffusion controlled^30–33^. However, of the two considered conditions, the un-annealed sample is more capacitive than the annealed one and to understand this behavior further, the electrochemical impedance of the samples was investigated via EIS.

**Figure 8:**
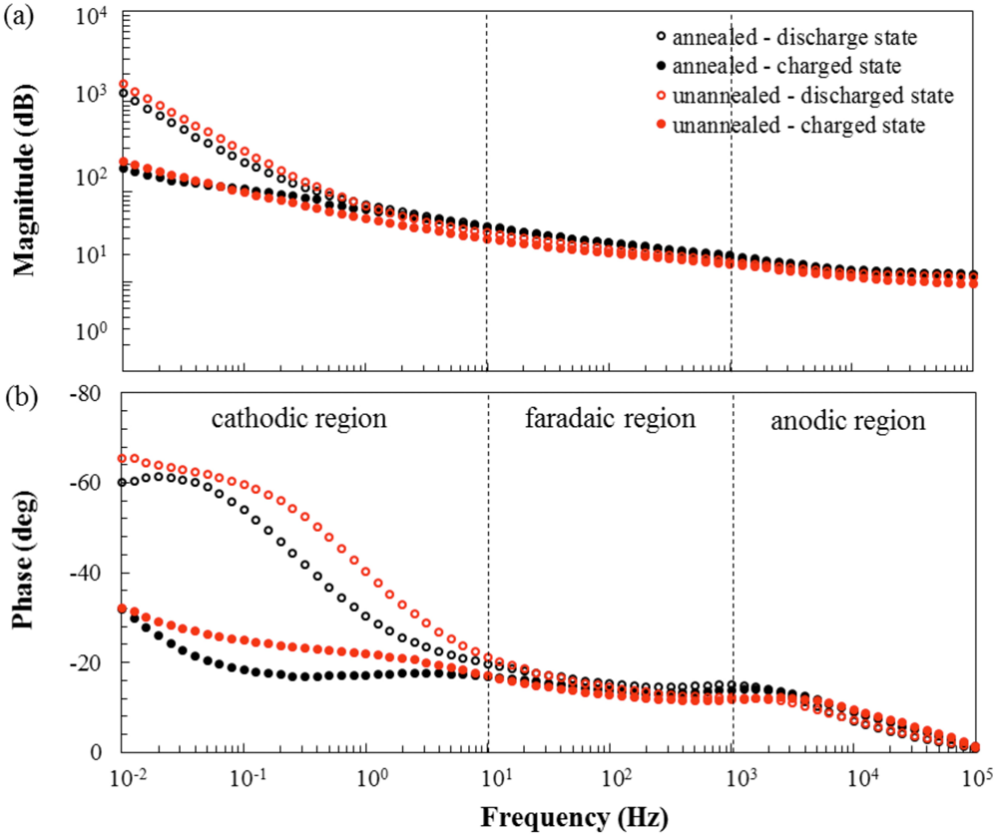
Bode magnitude and phase plots within the frequency range (0.01 to 10^5^) Hz.

As shown in Fig. 8, the Bode magnitude and phase plots of the samples can be designated into high, mid, and low-frequency regions. Thus, the high-frequency region corresponds to the anodic reaction (>10^3^ Hz). The mid-frequency region corresponds to the faradaic reaction (10^1^–10^3^) Hz. The low-frequency region corresponds to the cathodic reaction (10^−2^–10^1^) Hz. It can be observed that of the three regions, the cathodic region displayed an obvious difference in magnitude and phase which could be the consequence of annealing the MnO_2_ cathode. Herein, the annealed sample demonstrated lower values of magnitude and phase in comparison to the un-annealed one. On the other hand, fitting of the Nyquist plot using the equivalent circuit model (Fig. S7 and Table S4) showed that the annealed sample (139.8 $\Omega$) had lower resistance in the cathodic region than the un-annealed one (171.8 $\,\Omega$). This implied therefore that indeed the conductivity of the cathode was enhanced after annealing treatment through the reorientation of the δ-MnO_2_ sheets to form a well-ordered stacking.

## Conclusion

A novel binder-free orthorhombic δ-MnO_2_-C composite electrode was successfully synthesized via hydrothermal method. The strategy enabled the direct growth of interconnected δ-MnO_2_ particles with crumpled sheets-like morphology on the surface of the carbon fiber substrate. The as-synthesized (un-annealed) sample exhibited a high stacking disorder: namely, turbostratic stacking, of the δ-MnO_2_ sheets. Annealing of the δ-MnO_2_ facilitated the rearrangement of the δ-MnO_2_ sheets, thus forming a well-ordered structure, as indicated by the SAED hexagonal single crystal pattern, in comparison to the polycrystalline (ring) pattern of the un-annealed sample. Annealing of the orthorhombic δ-MnO_2_ showed an enhancement in δ-MnO_2_ sheets ordered stacking, surface area, and conductivity of the active material. Consequently, this produced efficient charge transfer as well as improved cyclability. Overall, better electrochemical performance of MnO_2_ as cathode material for DES based Zinc-ion battery (ZIB) was noted.

## Supplementary information

Supplementary Information

## Data Availability

The authors declare that all relevant data are within the paper.
