# Supplementary material for: Annealing induced a well-ordered single crystal δ-MnO2 and its electrochemical performance in zinc-ion battery
Source: Sci Rep. 2019 Oct 22;9:15107. doi: 10.1038/s41598-019-51692-x (PMC6805881; doi:10.1038/s41598-019-51692-x)
Supplement: Supplementary file 1 — Supplementary Information [file 41598_2019_51692_MOESM1_ESM.docx]

**Annealing induced a well-ordered single crystal δ-MnO_2_ and its electrochemical performance in zinc-ion battery**

Ryan Dula Corpuz^1,2^, Lyn Marie Z. De Juan^1,3^, Supareak Praserthdam^1,4^, Rojana Pornprasertsuk^5,6,7^, Tetsu Yonezawa^8^, Mai Thanh Nguyen^8^ and Soorathep Kheawhom^1,6,*^

^1^Department of Chemical Engineering, Faculty of Engineering, Chulalongkorn University, Bangkok, 10330, Thailand

^2^Department of Physics, Ateneo de Manila University, Quezon City, 1108, Philippines

^3^Department of Chemical Engineering, Faculty of Engineering, University of Santo Tomas, Manila, 1015, Philippines

^4^High-performance computing unit (CECC-HCU), Center of Excellence on Catalysis and Catalytic Reaction Engineering (CECC), Chulalongkorn University, Bangkok, 10333, Thailand

^5^Department of Materials Science, Faculty of Science, Chulalongkorn University, Bangkok, 10330, Thailand

^6^Research Unit of Advanced Materials for Energy Storage, Chulalongkorn University, Bangkok 10330, Thailand

^7^Center of Excellence in Petrochemical and Materials Technology, Chulalongkorn University, Bangkok 10330, Thailand

^8^Division of Materials Science and Engineering, Faculty of Engineering, Hokkaido University, Kita 13 Nishi 8, Sapporo, Hokkaido, 060-8628, Japan

*corresponding author: [soorathep.k@chula.ac.th](mailto:soorathep.K@chula.ac.th)

negative case

wave type spring

spacer

electrodeposited Zn on Ni foam

separator

MnO_2_ coated C-cloth

positive case

**Figure S1.** Schematic diagram of the Zn-MnO_2_ battery assembly.


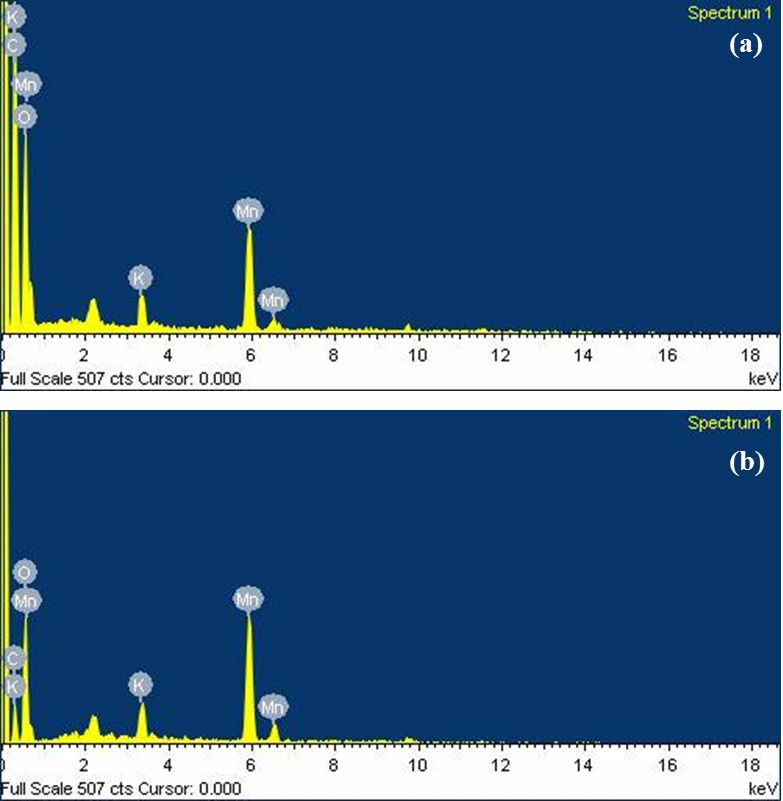

**Figure S2.** EDS analysis of (a) unannealed and (b) annealed sample, and (c) the amount of K^+^ per Mn atom for both annealed and unannealed sample.


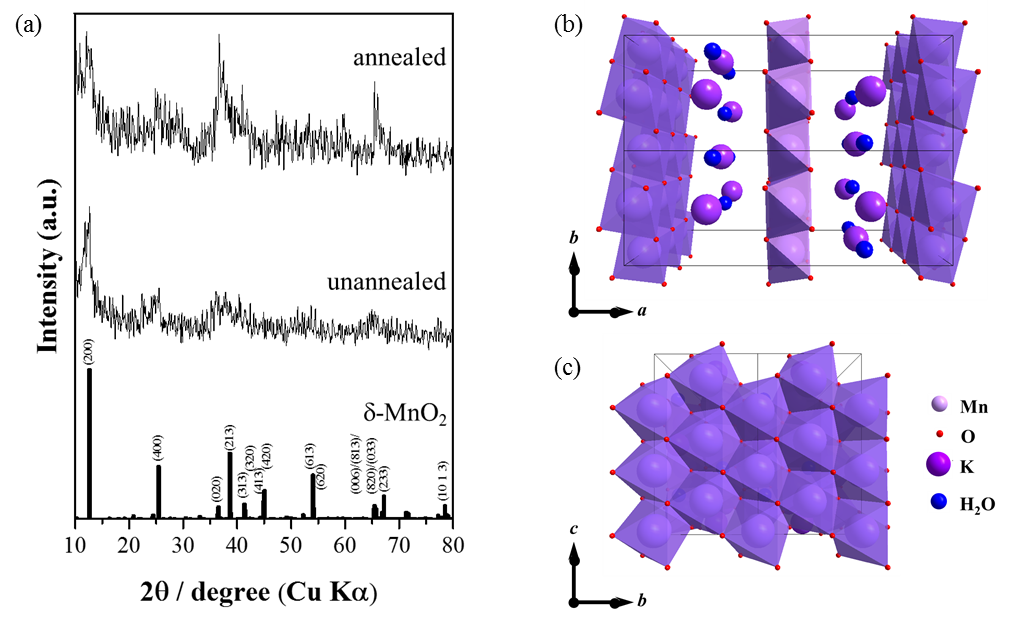


**Figure S3.** (a) XRD spectra of unannealed and annealed MnO_2_ with the corresponding calculated peaks of orthorhombic $\delta$-MnO_2_ (K_0.33_MnO_2_·0.66H_2_O)^[34]^ and (b,c) schematic diagram of orthorhombic $\delta$-MnO2 structure along the (b) {001} and (c) {100} planes.


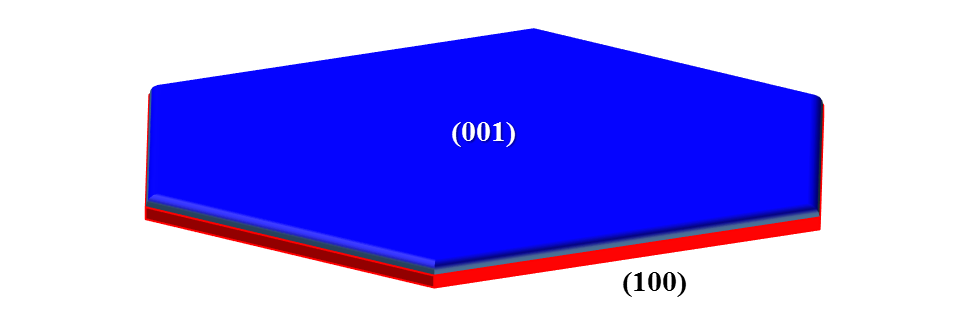


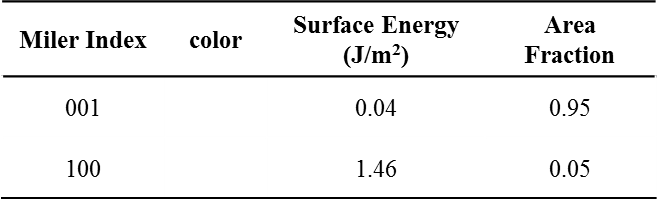


**Figure S4.** Wulff-constructed equilibrium shape of $\delta$-MnO_2_ with respect to surface energy^[34]^.

**Table S1.** BET isotherm and BJH pore pore-size distribution analysis of unannealed (turbostratic stacking) and annealed samples (well-ordered structure).

|  | **BET**  **Surface Area** | **BET**  **C*** | **BJH**  **Pore Volume** | **BJH**  **Pore radii** |
| --- | --- | --- | --- | --- |
|  | (m^2^ g^-1^) | (K_1_/K_L_) | (cm^3^ g^-1^) | (nm) |
| **Annealed** | 246.18 | 263.30 | 1.3188 | 6.03 |
| **Unannealed** | 106.32 | 160.05 | 0.7953 | 5.27 |

* C is the ratio between K_1_ and K_L_. The equilibrium constants K_1_ and K_L_ corresponds to the single molecule adsorbed per vacant site and the saturated vapor liquid equilibrium respectively.


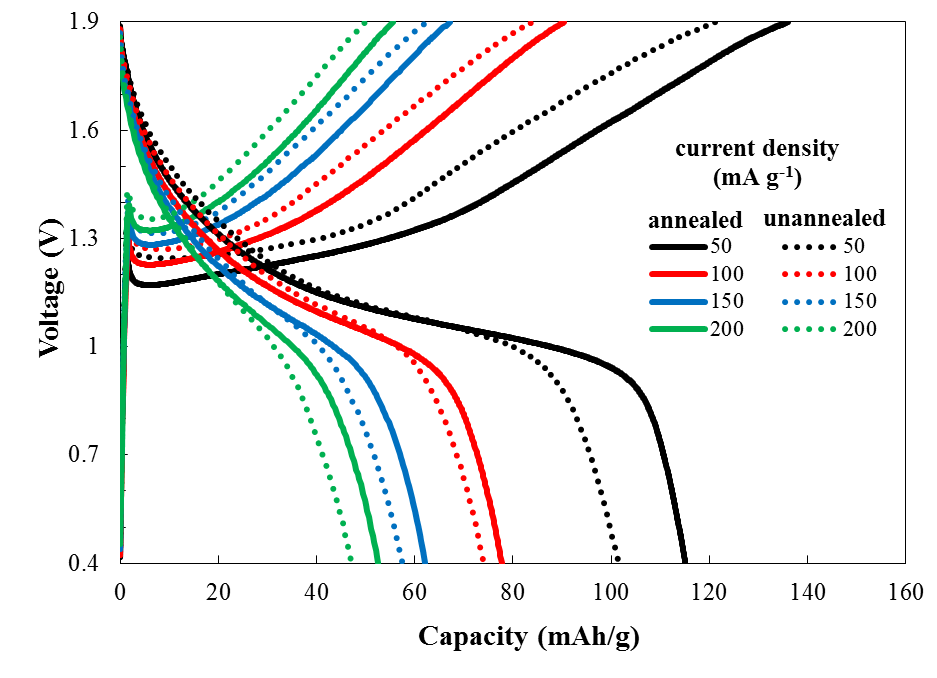


**Figure S5.** Galvanostatic charge-discharge profile of unannealed and annealed samples obtained within potential window: 0.4 - 1.9 V.

**Figure S6.** Cycle test of unannealed and annealed samples from 51^st^ to 100^th^ cycle. The hollow and filled circles correspond to charge and discharge values respectively, while diamond corresponds to the coulombic efficiency data.

**Table S2.** Calculation of contribution ratio at different scan rate of the unannealed and annealed samples.

|  | **Contribution Ratio (%)** | | | |
| --- | --- | --- | --- | --- |
| **scan rate** | **diffusion controlled** | | **capacitive effect** | |
| **(mV/s)** | **annealed** | **unannealed** | **annealed** | **unannealed** |
| 0.1 | 81.534 | 79.94 | 18.47 | 20.06 |
| 0.2 | 75.741 | 73.81 | 24.26 | 26.19 |
| 0.5 | 66.383 | 64.06 | 33.62 | 35.94 |
| 1.0 | 58.269 | 55.75 | 41.73 | 44.25 |
| 2.0 | 49.681 | 47.12 | 50.32 | 52.88 |

**Table S3.** Calculation of peak current density at different scan rate of unannealed and annealed samples.

| **scan rate** | **\|peak current (mA)\|** | | **\|peak current density (mA/g)\|** | |
| --- | --- | --- | --- | --- |
| **(mV/s)** | annealed | unannealed | annealed | unannealed |
| 0.1 | 0.131 | 0.105 | 52.41 | 42.01 |
| 0.2 | 0.211 | 0.179 | 84.26 | 71.54 |
| 0.5 | 0.434 | 0.348 | 173.57 | 139.21 |
| 1.0 | 0.642 | 0.541 | 256.78 | 216.31 |
| 2.0 | 1.000 | 0.843 | 400.18 | 337.20 |

** Peak current (mA) was obtained from the reaction on the cathode, i.e. reduction peak.*


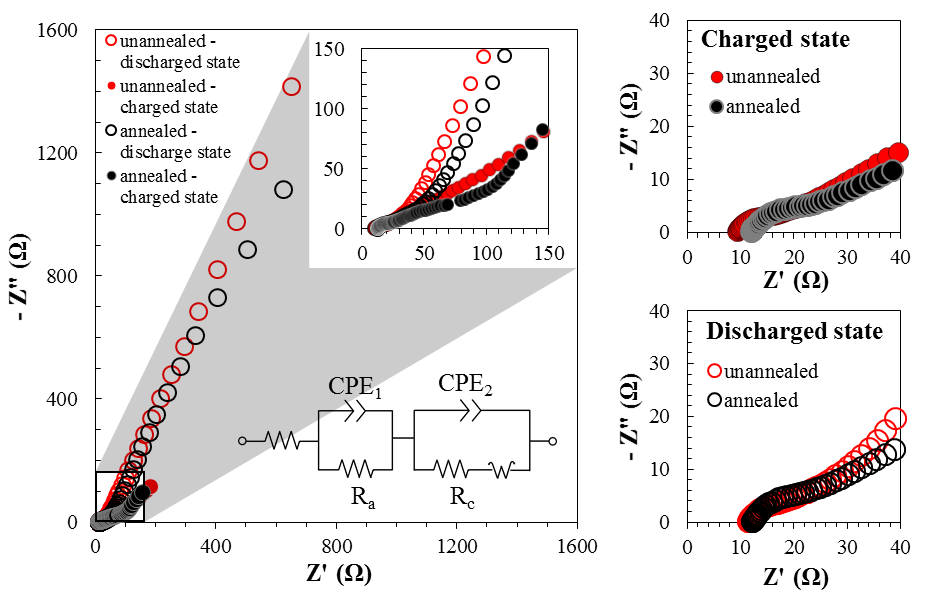


**Figure S7.** Nyquist plot of annealed sample after galvanostatic charge-discharge cycles.

**Table S4.** Calculated resistances for solution, anode, and cathode of unannealed and annealed samples.

|  | **Annealed**  **(Ω)** | **Unannealed**  **(**$\boldsymbol{\Omega}$**)** |
| --- | --- | --- |
| **R_s_** | 10.94 | 10.35 |
| **R_anode_** | 44.46 | 29.84 |
| **R_cathode_** | 139.8 | 171.8 |
|  |  |  |


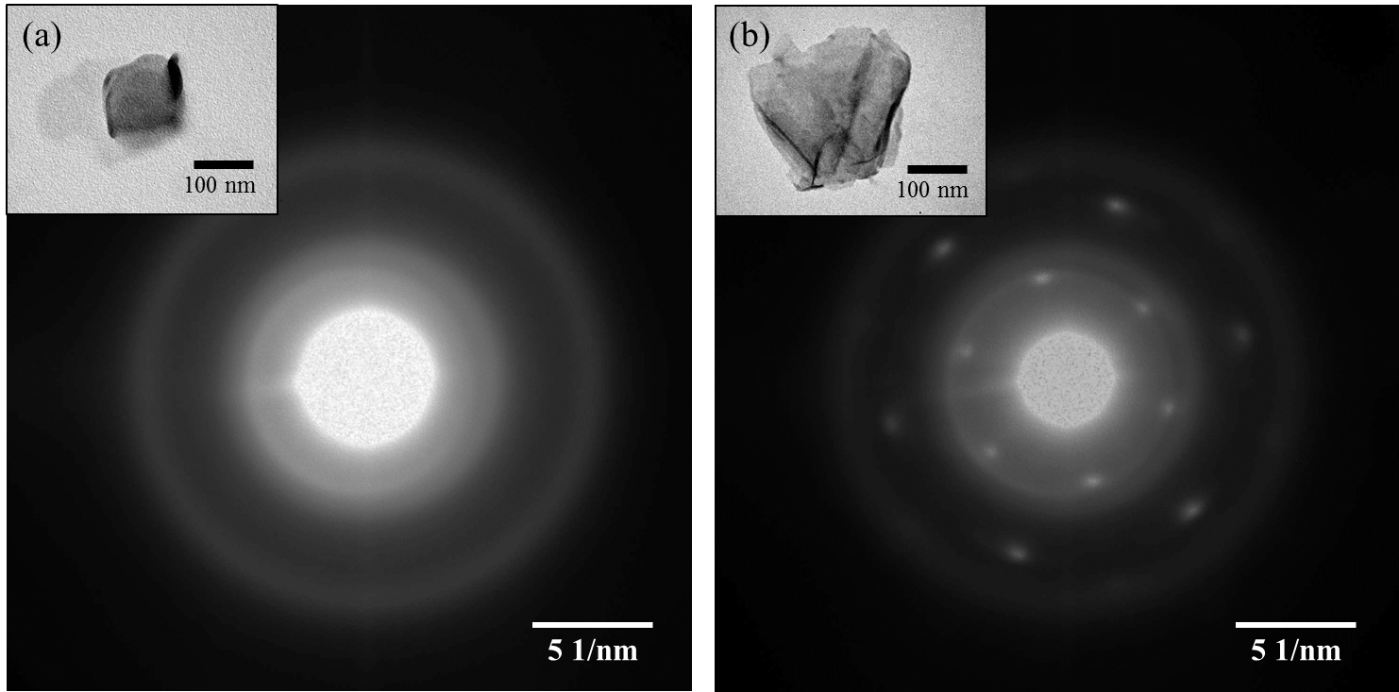


**Figure S8.** SAED patterns of unannealed and annealed samples with inset of the TEM images.


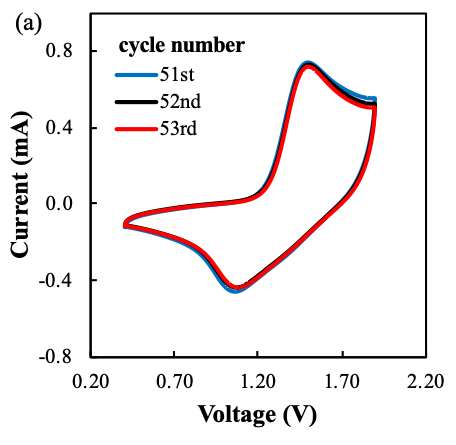

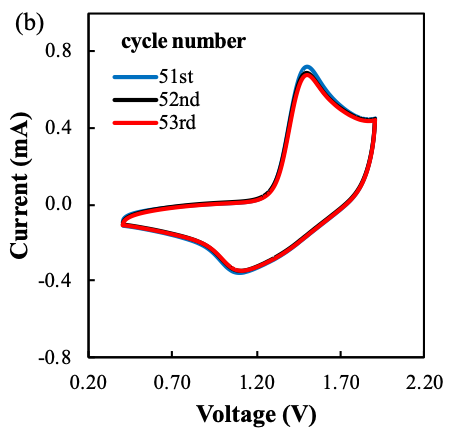


**Figure S9.** cyclic voltammogram of (a) annealed and (b) unannealed samples after the 50^th^ cycle of Galvanostatic charge discharge.
